# Supplementary material for: Global hypo-methylation in a proportion of glioblastoma enriched for an astrocytic signature is associated with increased invasion and altered immune landscape
Source: eLife. 2022 Nov 22;11:e77335. doi: 10.7554/eLife.77335 (PMC9681209; doi:10.7554/eLife.77335)
Supplement: Figure 2—source data 1. [file elife-77335-fig2-data1.zip › Figure_2_source_data_1/Figure_2I_J/homerResults/motif5.similar.html]

motif5

## Information for motif5

A
C
G
T
A
G
T
C
C
G
T
A
A
C
T
G
A
T
C
G
C
T
G
A
A
C
T
G
A
C
T
G
  
Reverse Opposite:  

A
G
T
C
A
G
T
C
G
A
C
T
A
T
G
C
G
T
A
C
A
C
G
T
A
C
T
G
C
G
T
A
  

|  |  |
| --- | --- |
| p-value: | 1e-48 |
| log p-value: | -1.114e+02 |
| Information Content per bp: | 1.925 |
| Number of Target Sequences with motif | 52.0 |
| Percentage of Target Sequences with motif | 7.16% |
| Number of Background Sequences with motif | 2.0 |
| Percentage of Background Sequences with motif | 0.70% |
| Average Position of motif in Targets | 101.8 +/- 59.2bp |
| Average Position of motif in Background | 108.0 +/- 0.0bp |
| Strand Bias (log2 ratio + to - strand density) | 0.4 |
| Multiplicity (# of sites on avg that occur together) | 1.08 |
| Motif File: | file (matrix) reverse opposite |

### Similar de novo motifs found

|  |  |  |  |  |  |  |  |
| --- | --- | --- | --- | --- | --- | --- | --- |
| Rank | Match Score | Redundant Motif | P-value | log P-value | % of Targets | % of Background | Motif file |
| 1 | 0.624 | C T G A C T A G T A C G C G T A C T A G T C A G T G C A C T G A C A T G C A G T C T A G A T G C | 1e-37 | -87.406215 | 6.06% | 0.61% | motif file (matrix) |
| 2 | 0.632 | T C A G C T G A C T A G T C A G C T G A C T A G T C A G T C G A T A G C G C T A T A G C G C T A | 1e-13 | -31.780026 | 5.10% | 1.37% | motif file (matrix) |
